# New Orleans School of Medicine

**Published:** 1856-09

**Authors:** 


### NEW ORLEANS SCHOOL OF MEDICINE.

We would desire to call attention to the advertisement of the "New Orleans School of Medicine" to be found upon the cover of this number. We learn that their new building, large enough to seat comfortably 250 Students, will soon be completed, and everything will be ready for the Lectures, at the time specified. Most of the Faculty are now visiting physicians, and the Professor of Surgery is Resident Surgeon at the Charity Hospital, and the Board of Administrators having allowed them the free use of that Institution during the Course of Lectures, they certainly have it in their power to offer superior advantages.

